# Description of a New Bed and Bedstead for the Use of Insane and Other Patients

**Published:** 1852-07-01

**Authors:** William Wood

**Affiliations:** LICENTIATE OF THE ROYAL COLLEGE OF PHYSICIANS, AND MEDICAL OFFICER OF BETHLEM HOSPITAL


### DESCRIPTION OF A NEW BED AND BEDSTEAD FOR THE USE OF INSANE AND OTHER PATIENTS.

BY WM. WOOD, M.D., LICENTIATE OF THE ROYAL COLLEGE OF PHYSICIANS, AND MEDICAL OFFICER OF BETHLEM HOSPITAL.

In the treatment of insanity, as, indeed, in the preservation of health under any circumstances, the importance of sufficient sleep can scarcely be over-estimated. It is not less essential than food, and is frequently more difficult of attainment than the ensuring of a proper supply of nourishment. To woo "nature's soft nurse" successfully, we must be careful to provide a suitable resting-place for the body agitated by the fearful forebodings and wild imaginations of a troubled mind. Nothing is so well calculated "to steep the senses in forgetfulness" as ensuring the comfort and rest of the worn frame; and we all know how great the prospect of obtaining sleep is diminished by an uncomfortable bed. For the ordinary class of patients the usual bedding is all that is required; but those who, from some particular propensity, inability, or infirmity, increase their personal discomfort by ceasing to pay any regard to cleanliness, and so acquire habits which are prejudicial to their general health, some other provision is necessary. I know it is said that there need not be any dirty patients, and that proper management and attention will ensure the cleanliness of all; but as far as my experience goes, this, like every other rule, is liable to exceptions. For instance, taking the male side of this hospital, there are at this moment four patients who are entirely regardless of personal cleanliness, consequently are frequently dirty, particularly at night; and this is about the usual average. There are generally about three more who are very frequently wet. These cases almost invariably occur among the curable class of patients, or perhaps, more strictly speaking, in those whose malady is recent; for of these seven but one belongs to the incurable class, and six to the curable. Although a certain proportion of the wet and dirty patients are those whose minds are too much disturbed to guide their actions and control their conduct, there are others who are actively and determinedly mischievous, but have yet sufficient intelligence to derive positive pleasure from the trouble they give—

sometimes absolutely rejoicing in it, and taunting their attendants with the disgusting duty they have imposed upon them. Others, again, too much absorbed in their insane thoughts to heed the consequences of filthy habits, but appearing to derive a kind of pleasure from indulging in them—these cannot be reasoned with, for any impression that is made upon them is most evanescent, and ceases almost on the instant to exercise the smallest influence over them. Such cases must always occur, and while they exist in an acute form their ever-varying mental condition renders utterly abortive the attempt to establish the habit of cleanliness; when, however, the acute stage of the malady is past, patients become more the creatures of habit; for this reason, among others, that their mental condition is more permanently settled and less varying. For dirty patients, then, the beds most commonly in use are either of straw or stretchers—that is, strong canvas or sacking stretched tightly over a wooden frame. As regards the comparative comfort and healthiness of these two, my opinion is decidedly in favour of the straw bed; for independently of the wholesome aroma from clean straw, which I believe has a positively beneficial effect, any moisture runs away more readily than it can from the stretcher, into which in fact, it soaks until by capillary attraction a large surface of it is saturated. Then a straw bed, though far from soft, is softer than a tightly-stretched sacking, on which no bed of any kind is placed when used for wet and dirty patients. The straw bed, then, being softer, is more comfortable, and it is also warmer, inasmuch as the patient's body sinks more into it than it can when placed on the tense and therefore hard surface of a stretcher. For these reasons my impression is, that the straw bed is the better of the two; but to it there are serious objections, independently of the ideas with which such bedding is associated. Whatever may be the means adopted for enclosing the straw, mischievous patients will always destroy the covering, and thus a quantity of loose straw affords them the means of occupation, and such diversion of their thoughts as divests them of any idea of attempting to procure sleep, besides enabling them to scatter the whole contents of their bedding, rendered impure and offensive, all over the room.

To obviate these objections, I considered how a bed might be constructed which should be soft and yielding, but at the same time of sufficient strength to resist the attempts of the mischievous to destroy it, while it allowed any moisture to escape at once, without leaving a wet and offensive surface about the person of the patient. This object was effected by making an oak frame, about 6 feet 3 inches long by 3 feet wide; over this was stretched webbing and canvass to support a stuffing of horsehair, as in the ordinary seat of a chair or sofa. The stuffing, however, was so arranged as for the surface to present a gradual fall towards the centre, which was pierced by a small hole extending through the whole thickness of the bed thus constructed; and through this hole a brass tube, with an extended and flattened edge, somewhat resembling the neck and round flattened lip of a decanter, was passed, a screw being cut on the outside of the lower part of the tube, which projected on the under surface of the bed, and was secured in its place by a nut, which so assisted in making the depression in the centre by approximating, in proportion to the tightness with which it was screwed up, the upper and under surfaces of the bed, at the same time that it brought the under surface of the extended lip of the tube in close contact with the upper surface of the bed immediately surrounding the hole, so as to prevent any moisture getting down by the side of the tube into the inside or stuffing of the bed. Before, however, fixing this tube, the whole of this bed was covered with a sort of sailcloth, made expressly by Messrs. Edgington, strong and perfectly waterproof, the edge, of course, being pierced for the tube already described, and the outside edges secured by being brought over the frame and nailed to its under surface, so that no point presented which could be taken hold of by a mischievous patient. A frame thus stuffed is easily held in its place in a box bedstead by a very simple fastening, consisting of a screw worked by a small key, as shown in the new bedstead which I have since had made, and which I shall describe presently. It will be understood, then, that the bed just described consists of a sort of frame mattress, made so as to present a gradual fall from both ends and sides towards the centre, so that any wet would naturally fall to this spot, and escape through the tube, the whole upper surface of the bed being covered with a strong waterproof material. A bed of this description has now been in use in this hospital for about twelve months, during which time it has been severely tested, but has perfectly answered the purpose for which it was made. It is essential to the successful employment of this bed that the top surface should be perfectly waterproof and strong; and when these conditions are fulfilled, I believe it will be found that this bed will be a very great comfort in the cases of patients, whether insane or not, whose infirmities or more active disease make it extremely difficult to keep them clean. A bed on this principle, however offensive it may be made by the matters discharged upon it, may in a few moments be rendered perfectly sweet, and again fit for immediate use. When the patient is not mischievously disposed, of course no fastening is necessary, and the bed may be simply laid on an ordinary bedstead, the only thing required being a provision for the escape of any fluid passing through the central tube. Another important consideration is, that this bed may be made for a very few shillings more than an ordinary horse-hair mattress.

A sketch of it is shown in the accompanying drawing:—Fig. 1.

The circumstances which led me to contrive what I have called the "Enclosed Bed," will be best explained by a brief sketch of the case for which it was made.

A patient was admitted towards the latter end of last year in a condition approaching dementia, which appeared to have been brought on by great mental anxiety; he was generally quiet and silent during the day, and almost helpless, being so much confused in all his acts as not to know the different articles of dress from one another, and would try to put on his coat as trousers, and the like; his memory was entirely gone, and he seemed to have no idea of the lapse of time. He was very dirty in his habits and constantly noisy at night, it being his uniform practice to get out of bed, and stand and shout, with a power, of which, from his appearance, nobody would have supposed him capable. This went on for several weeks, and so far from any impression being made upon him, he seemed to get, if possible, worse and more noisy, so that the patients in a distant part of the building were roused from sleep by him, to say nothing of his immediate neighbours, who were seriously disturbed, while he himself suffered from the want of rest and sleep. It occurred to me that some means might be adopted, without resorting to any of the usual expedients for fastening him bodily to the bed, to prevent his getting out of bed; and as the patient happened to have a relation also a patient in the hospital at the same time, who was a cabinet-maker, I set him to work to carry out my idea, which, in fact, consisted of attaching to a sort of crib bedstead a kind of lid, formed of network of webbing, stretched on a frame. This was at once commenced, and made entirely by the relative of the patient, who took a very great interest in the experiment, had the satisfaction of seeing it succeed perfectly, and soon after left the hospital well. In my notes of the case the following memorandum occurs:—

January 22nd,—"Slept for the first time last night in the enclosed bed, and passed a very tranquil night; indeed, from the appearance of the bed this morning, he seems scarcely to have moved during the night. With one exception, he has been reported every night in the watchman's book since 13th Dec., for being out of bed and noisy, and his noise has been such as to wake patients in the criminal wing, which is a perfectly distinct and separate building, some distance off."

He continued to sleep in this enclosed bed without ever making any noise, from the night he was first put into it until April 11th, when, believing that the habit of sleeping quietly had become sufficiently established, I directed him to be put in an ordinary bed, and found that he now slept as well there as he had done in the enclosed bed, and from that time forward continued to do so without ever again making any noise.

Besides such cases as that which I have thus briefly sketched, there are many others, including some epileptics, in which such a bed would be most useful and convenient at the same time that it is safe, and does not at all interfere with the ordinary movements of a person in bed, although it effectually prevents his getting out, as will be seen by the accompanying drawing. This contrivance consists of a sort of crib bedstead, the inside of which all round should be padded and covered with the same waterproof covering as the bed already described; this would, in the case, for instance, of an epileptic patient, prevent his doing himself injury by his own violence; and besides, it can be so easily cleaned; then the thickness of the frame, which serves the purpose of a lid, must correspond exactly to the thickness of the sides of the crib as seen at 5. 5. 5., so that no wood-work projects over the patient, and, indeed, nothing but the net-work of webbing, which must be of the strongest kind, two thicknesses of it being firmly stitched together, particularly at every crossing. This lid is secured behind by three strong hinges, one limb of each extending down the side, or rather the back of the crib, on the outside, and the other turning over the top edge of the frame, so as to hold it securely in front; it is secured by means of the bar or flap, which is attached to the front edge of the frame by spring hinges, which keep it at right angles with the frame, and, when shut down, parallel with the front side of the crib. The object of the spring hinges is to keep this flap pressed against the front side of the crib, when shut in such a manner that the fixed catches or bolts, which project from its inner surface, may be drawn into and retained in the holes of the plates, 2, 2, 2. These plates, by being turned over the front edge of the crib, answer the purpose of striking plates, and, as the lid is shut down, guide the projecting bolts of the flap into the openings destined to receive them, the spring hinges allowing the flap to yield sufficiently for these bolts to slip over the edges of the plates, and then pulling the flap inwards, so as to answer the purpose of a spring lock, which, when applied to a door, is locked by simply pulling the door to. It will be obvious then that all that is necessary to open the lid, when thus secured, is to draw away the flap from the front of the crib, and in doing so the three bolts are drawn out of the openings 3. 3. 3. in the plates 2. 2. 2. A patient might do this himself by putting his hand through the opening of the webbing, and pulling, or tilting up the flap; to prevent this, I had a simple fastening attached, which consists of a screw seen at 1. on the flap, which is retained in its place by means of a shoulder, and can only be protruded or withdrawn by means of a key, a few turns of which would screw it into the plate 4. shown on the front of the crib at the centre; this could only be undone by a few turns of the key in the opposite direction; the part of the screw on which the key fits being buried in the thickness of the flap, and therefore out of the reach of fingers, and only got at by a key.

The principle of this bedstead, then, is that of a crib with a lid to it, the inside being padded; the bedding being either the new bed which I have described above, or ordinary mattresses, the lid consisting of a net-work of webbing, without any woodwork projecting over the patient as he lies in bed, and being at a sufficient height from the top of the mattress to allow of free movements by turning from side to side, without touching the cross-webbing of the lid.

